# Diversification and intensification of agricultural adaptation from global to local scales

**DOI:** 10.1371/journal.pone.0196392

**Published:** 2018-05-04

**Authors:** Minjie Chen, Bruno Wichmann, Marty Luckert, Leigh Winowiecki, Wiebke Förch, Peter Läderach

**Affiliations:** 1 Faculty of Agricultural, Life & Environment Sciences, University of Alberta, Edmonton, Alberta, Canada; 2 The World Agroforestry Centre (ICRAF), Nairobi, Kenya; 3 Gesellschaft für Internationale Zusammenarbeit (GIZ), Gaborone, Botswana; 4 Decision and Policy Analysis (DAPA), International Center for Tropical Agriculture (CIAT), Hanoi, Vietnam; Wageningen University, NETHERLANDS

## Abstract

Smallholder farming systems are vulnerable to a number of challenges, including continued population growth, urbanization, income disparities, land degradation, decreasing farm size and productivity, all of which are compounded by uncertainty of climatic patterns. Understanding determinants of smallholder farming practices is critical for designing and implementing successful interventions, including climate change adaptation programs. We examine two dimensions wherein smallholder farmers may adapt agricultural practices; through intensification (i.e., adopt more practices) or diversification (i.e. adopt different practices). We use data on 5314 randomly sampled households located in 38 sites in 15 countries across four regions (East and West Africa, South Asia, and Central America). We estimate empirical models designed to assess determinants of both intensification and diversification of adaptation activities at global scales. Aspects of adaptive capacity that are found to increase intensification of adaptation globally include variables associated with access to information and human capital, financial considerations, assets, household infrastructure and experience. In contrast, there are few global drivers of adaptive diversification, with a notable exception being access to weather information, which also increases adaptive intensification. Investigating reasons for adaptation indicate that conditions present in underdeveloped markets provide the primary impetus for adaptation, even in the context of climate change. We also compare determinants across spatial scales, which reveals a variety of local avenues through which policy interventions can relax economic constraints and boost agricultural adaptation for both intensification and diversification. For example, access to weather information does not affect intensification adaptation in Africa, but is significant at several sites in Bangladesh and India. Moreover, this information leads to diversification of adaptive activities on some sites in South Asia and Central America, but increases specialization in West and East Africa.

## Introduction

Smallholder farming systems, and hence food security, are vulnerable to a number of challenges, including continued population growth, urbanization, income disparities, land degradation, and decreasing farm size [1, 2, 3]. Further challenging smallholder farming systems is climate change [4]. Extreme climate events, such as droughts and heavy rainfall, are becoming more frequent over the last decades, especially in areas such as Sub-Saharan Africa. These trends are likely to continue and highlight the need of a deeper understanding of how smallholder farmers in developing countries may respond to these changing circumstances [5].

Adaptation of farming practices has the potential to reduce the negative impacts of climate change [6]. The Intergovernmental Panel on Climate Change defines adaptation as “the process of adjustment to actual or expected climate and its effects seeking to moderate, avoid harm, or exploit beneficial opportunities” (see page 118 [7]. Though climate change is arguably a key stimulus for adaptation, we recognize that a number of other drivers could also be stimulating change, such as market conditions, pests and disease, government programs, and the availability of labor and land. Therefore, our analysis is undertaken within the context of multiple drivers of change. Our approach, to consider multiple drivers at a global scale, allows us to estimate the relative importance of the various general reasons for change, which also controls for these causes of change (as fixed effects) with respect to their influence on adaptation.

Processes of adjustment are poorly understood and, unfortunately, are frequently constrained by socio-economic characteristics that constitute elements of adaptive capacity. As one of the landmark conclusions of COP21 (the 2015 U.N. Climate Change Conference in Paris), the international community agreed to establish a global goal of enhancing adaptive capacity, strengthening resilience and reducing vulnerability to climate change [8]. The successful pursuit of such a goal relies heavily on understanding how and why humans adapt. Following this imperative, the overall goal of this paper is to contribute to our knowledge regarding the adaptive behavior of smallholder farmers.

In pursuit of our goal, our first contribution is to present global results reflecting a two-dimensional examination of agricultural adjustment processes; adaptation by intensification of farming activities (i.e. do more things), and adaptation by diversification of farming activities (i.e. do different things).

Global efforts to invest in agricultural development, including the Maputo Declaration [9], often rely on intensification strategies to cope with rising food demand. In contrast, diversification is an alternative adaptive strategy to respond to fluctuating markets and climate. Intensification and diversification strategies are often already on-going activities on the farm. But these strategies may change in response to changing environments, thereby triggering adaptation. A better understanding of determinants of agricultural intensification and diversification is likely to yield better programs to develop increased resilience in farming systems in the presence of changing climates [10].

A key issue in understanding adaptation is its measurement. Several studies have investigated determinants of adaptation by treating adaptation as a binary choice (i.e. the farmer adapts or does not). These studies investigate the extensive margin of adaptation by either focusing on a single adaptive activity [11] or a set of activities measured by a single outcome [12]. Another set of studies investigate the determinants of adaptation at the intensive margin (i.e. how much to adapt). Intensity of adaption is typically measured by the number of adaptation activities undertaken by a farmer [13, 14, 15, 16]. There is another group of studies which have investigated whether adaptation improves aspects of welfare of households. For example investigate impacts of adaptive strategies [14], to adapt to long-term changes in temperature and rainfall, on households’ net revenues in the Nile Basin of Ethiopia. Former studies [17, 18] use simulated climate data to project future crop productivity based on different adaptation strategies for, respectively, Switzerland and the world. Another publication [19] investigates whether increased counts of adaptive activities leads to increases in food security. In this paper, we focus our efforts on determinants of adaptation without considering links to measures of welfare.

In addition to considering what drives the extensive and intensive margins of adaptation, another relevant dimension is whether diversified adaptive activities are undertaken. Diversifying adaptation strategies by implementing farming activities of different types (e.g. activities related to the management of existing crops vs changing crop varieties) may be a rational response to the rising frequency of unpredictable extreme weather conditions. Such strategies can help households to hedge against different sources of risk and to reduce vulnerabilities in an environment of rapidly changing climate.

Diversification of livelihood activities among smallholder farmers has a long history of research [20]. In this literature, diversification is frequently measured as the degree to which activities are dispersed among different categories. But investigating adaptive diversifying activities is a bit different, in that it involves studying to what extent *changes* in activities are diversified. Previous studies that have investigated diversification of adaptation activities have employed measures such as number of farm-level adaptation practices [21] or acreage-weighted income proportions from different crop types [22, 23]. In order to investigate the types of adaptive activities being undertaken, some studies have included categories of counts of adaptive activities in their analyses [16, 18].

This study takes a different approach to capture diversification of adaptation: the Herfindahl-Hirschman index (HHI). This index is widely used in the industrial organization literature to quantify market power [24, 25, 26] and in the agricultural/development literature to measure income diversification [24, 25, 27, 28]. Specifically, we develop an HHI for agricultural diversification by classifying adaptation activities in three mutually exclusive categories of farming practices (crop management, crop varieties, and soil, water and land management). A farmer’s adaptation strategy is perfectly diversified if he adopts a third of his farming activities in each of the three categories. To our knowledge, no previous work has studied diversity in adaptation activities using measures of dispersion, such as the HHI.

The omission of diversification measures has potentially serious consequences for our understanding of adaptation, because we hypothesize that determinants of adaptation intensity could differ from those that drive adaptation diversification. On one hand, governments and donor agencies may design policies to facilitate the implementation of a particular set of adaptive activities, therefore improving adaptation intensity. This policy, however, may reduce the diversification of adaptation and result in greater (unintended) risk exposure. On the other hand, policies targeted to improve the diversification of adaptation may dilute adaptation efforts causing a decrease in intensification. More optimistically, it is also possible that there are synergies between intensification and diversification, and policies that relax the constraint on certain elements of adaptive capacity may be beneficial to both intensity and diversification dimensions of adaptation. Following the need to clarify these relationships, we compare and contrast determinants of adaptation intensity and adaptation diversification.

The second contribution of the paper is to explore possible differences between global and local determinants of the two dimensions of agricultural adaptation. Global drivers are those elements of adaptive capacity that are expected to influence adaptation across different regions of the developing world. In contrast, local drivers are elements that may be used to leverage adaptation at specific sites, but are not necessarily instrumental in promoting adaptation around the globe.

Most studies in the agricultural adaptation literature have focused on single scales of analysis. For example, studies have been conducted at the sub-national level [15, 23, 29], at the country level [13, 30], at a regional level [18, 31, 32] and at the international level [21]. Such studies frequently employ fixed effects to control for geographical differences that may occur at lower levels, such as study sites. But none of these studies have investigated their data to see whether the same determinants that arise at higher scales are also key determinants at more local scales.

Economists, and other applied scholars, are generally interested in working with the largest possible sample to increase statistical power of econometric models in pursuit of econometric identification of generalizable central tendencies. Less work has been done in assessing differences between multi-scale analyses. To what extent do global assessments of drivers of adaptation strategies hold at local scales? A recent study [33] argues that adaptation is an issue relevant at local, national and international levels. Effective adaptation strategies need to meet the objectives of adaptation at different scales. For example, global market shocks have important effects on local agriculture and have been shown to affect production and land use [34, 35]. In some cases, local economic conditions can have significant effects in global agricultural markets [36]. The complex relationship between local and global economies goes beyond markets into climate change. Global climate change converges in localities, and changes that happen at a local scale contribute to global change. In turn, local communities are affected by global climate patterns [37]. Adaptation strategies can be sensitive to spatial scales that feature diverse socio-economic, biophysical, environmental and institutional characteristics. Effective adaptation depends on policies and measures that are coordinated across international, regional, national and sub-national levels, and thus understanding the driving factors of adaptation across multiple scales is essential to inform programs design and implementation by governments and donor agencies. Based on this gap in the literature, our second contribution is to compare determinants of adaptation (both intensity and diversification measures) at global and local scales.

The global scope of our analysis creates both strengths and challenges. Some scholars, such as Hinkel [38], have concluded that the specific conditions needed for the rigorous analysis of adaptation requires in-depth localized knowledge. But policy makers could greatly benefit from more generalizable results that could be supported by broad-based policies. Investigating adaptation at various scales could potentially disclose such generalizable results, but perhaps at a cost of a loss of rigor in understanding adaptation processes. Along these lines, our study offers results that disclose information about this trade-off between generalizability and rigor, thus contributing to the debate about external and internal validity in empirical research.

In pursuit of our objectives, we analyze a unique global dataset collected by CCAFS [39]. We describe this dataset in the next section. Section 3 describes our methods, including the measures of adaptation and how they are related to determinants of adaptation through empirical models of adaptation intensity and adaptation diversification. Section 4 presents the adaptation levels found by the study and discusses the results of the two empirical models estimated at the global scale, and compares the determinants of adaptation at global and local scales. We conclude with a discussion of the implications of our findings.

### Data, study sites, and sampling

This study uses cross-sectional household level data from the CCAFS Baseline Household Survey [40, 41]. The survey collected information about food security, household assets, agricultural production/selling diversity, farming practices adaptation, climate information and mitigation behaviors, and gender differences in agricultural activities [16, 41].

**Fig 1:**
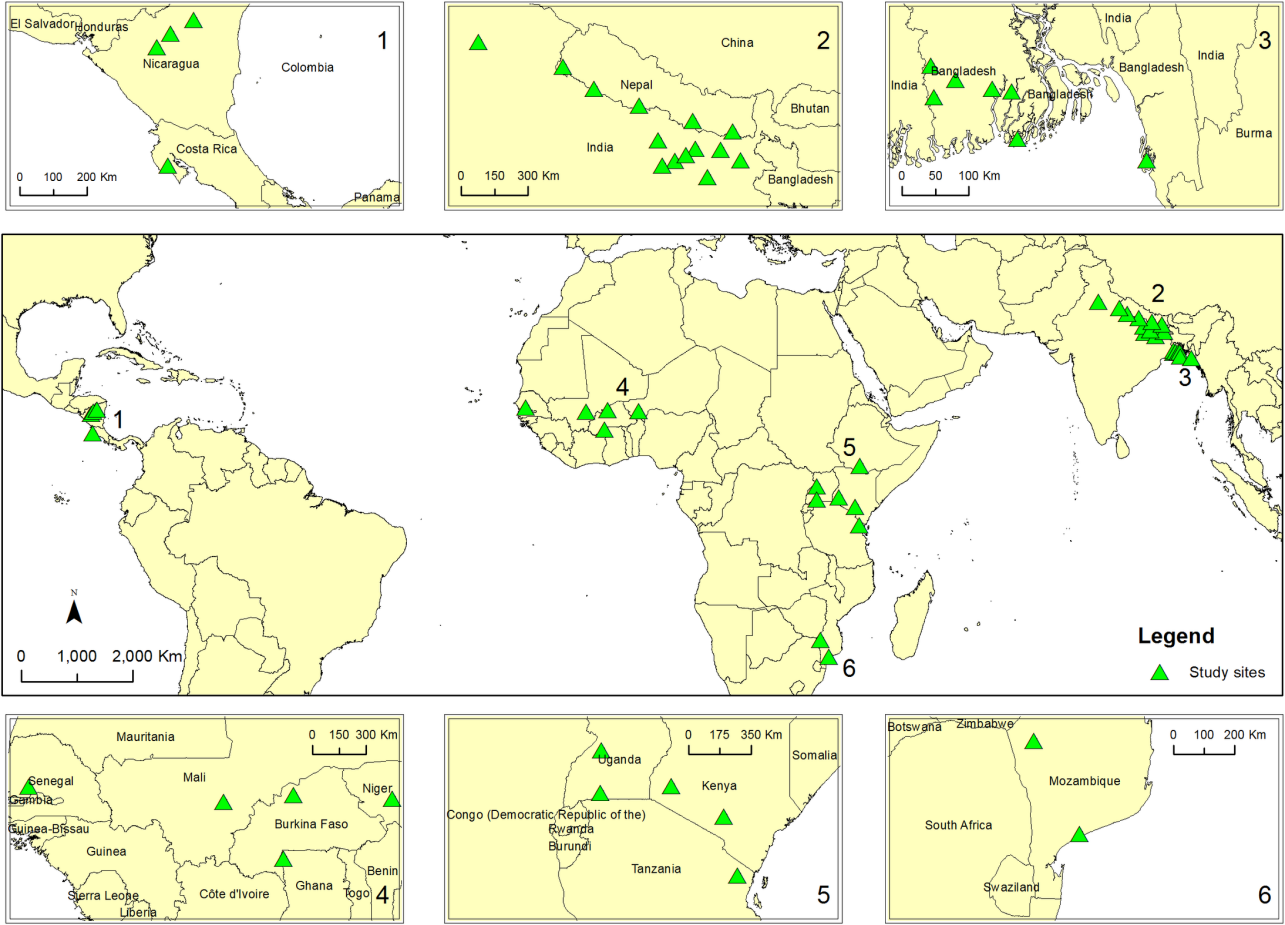
Location of the study sites (green triangles).

Data were collected from households located in four global regions: West Africa, East Africa, South Asia and Central America, from late 2010 to late 2013 for the Africa and Asia sites and 2014 for the Central America sites. These regions were selected for having high levels of poverty and vulnerability, significant and diverse climate challenges and opportunities for policy or project interventions [42]. The CCAFS survey team collaborated with regional and national partner organizations to choose the study sites in selected regions and to support data collection and analyses [43, 44]. Sites selected for the survey (see Fig 1) in general share several key features with the respective regions/countries, such as the biophysical and agro-ecological gradients, and socio-economic and demographic characteristics. An initial study [45] provides some analyses of the CCAFS household baseline data, while site characteristics can be found in a series of site atlases (https://ccafs.cgiar.org/atlas-ccafs-sites).

Household sampling in the selected sites was initially done by specifying a randomly located 10x10 km sampling frame in each site; while 30x30km sites were selected in West Africa and Ethiopia due to low population densities. Then, seven villages located in the sampling frame and 20 households in each village were randomly selected for conducting the survey. When the number of villages in a block was less than or equal to seven, then all villages were selected for the sampling of households [46]. Our dataset consists of 5,314 randomly sampled households located in 38 sites in 15 countries across the four regions. Table 1 summarizes the spatial distribution of households in our sample.

**Table 1:** Study sites information of CCAFS baseline household survey.

Region	Country	Number of Sites	Number of Households
West Africa	Ghana	1	140
	Burkina Faso	1	140
	Mali	1	141
	Niger	1	140
	Senegal	1	138
	Mozambique	2	280
East Africa	Ethiopia	1	140
	Kenya	2	279
	Tanzania	1	140
	Uganda	2	280
South Asia	Bangladesh	7	980
	India	9	1260
	Nepal	5	697
Central America	Costa Rica	1	139
	Nicaragua	3	420
Total	15	38	5,314

## Methods

### Measuring two dimensions of adaptation

Our measures of adaptation (both intensification and diversification) are constructed using information from questions that ask about changes that were made regarding households’ farming activities within the past 10 years. Households were instructed to select all alternatives that would apply from a list of 46 farming practices (S1 Table). To measure adaptation intensity, and following the literature cited above, we count the total number of farming practice changes made by each household. The intensification measure of adaptation does not account for differences in the types of farming activities farmers have chosen to adapt.

We investigate a second dimension of adaptation, diversification, by examining the proportion of changes within each of three categories of farming activities: i) crop management, ii) crop varieties, and iii) soil, water and land management. These categories are constructed by categorizing the 46 adaptive activities contained in the survey instrument (see S1 Table). Similar categorizing approaches have been previously used [31, 46, 47]. In order to measure the diversification of adaptation activities for each household, we count the number of farming practices contained in each category and construct a normalized Herfindahl-Hirschman Index (HHI). This approach has been extensively used to study corporate performance involving diversification [48, 49, 50]. Its application in agriculture has also been explored with regard to the diversification of crops [24, 25]. Formally, the definition of the normalized HHI is:

$$HHI=\frac{\sum_{k=1}^{K}s_{k}^{2}-1/K}{1-1/K}$$ (1)

where *k = 1*,*2*,*3* indexes a category, *K = 3* is the total number of categories for our study, and *s*_*k*_ is the ratio between the number of farming practices applied in category *k* and the total number of farming practices applied in all categories.

Note that the set of farmers which we include in our diversification analysis is smaller than those included in our intensification analysis. While the intensification measure allows us to combine information on farmers that both adapt and do not adapt (i.e. the count of practices changed is greater than or equal to zero), the diversification measure is only defined for farmers that adapt.

### Empirical models

We estimate two forms of econometric models of adaptation; one to explain adaptation by intensification, and one to explain adaptation by diversification. Moreover, to compare determinants across spatial scales, each of these models is estimated at global and local scales. Global models include observations from all sites and households, while local models are restricted to observations of households at each site. In total we estimate two global models (i.e. one for intensity and one for diversification) and 76 local models (i.e. for each of 38 sites, we estimate a diversification and an intensification model).

Poor households in rural areas of developing countries face constraints regarding adaptation [51]. To account for these constraints, empirical models of adaptation often account for several socio-economic factors that act as elements of adaptive capacity. These determinants include variables that capture access to information, human capital, financial and physical assets, farm and household characteristics, and farming and climate crises experience [8, 34, 52, 53, 54]. The CCAFS survey provides us with a number of elements of adaptive capacity. It also offers information on the reasons for adaptation. The questions about reasons follow the adaptation question. So, first farmers were asked “What changes have you made in the way you have been managing your land, crops and farm animals over the last 10 years? Have you….”; based on a list of 46 farming practices (see S1 Table). Households were instructed to select all alternatives that would apply from this list. Next, farmers were asked “Why you have made these changes?” This question was followed by a list of reasons and households were instructed to select all alternatives that would apply. The S2 Table offers descriptive statistics of the elements of adaptive capacity and the reasons for change used in our empirical model.

We consider the following regression model of adaptation by intensification:

$$I_{isr}=\sum_{k}\beta_{k}X_{k,isr}+\sum_{m}\gamma_{m}R_{m,isr}+\lambda_{s}+u_{r}+\sum_{c}v_{c}+\varepsilon_{isr}$$ (2)

where *I*_*isr*_ is the adaptation intensity of household *i* located in site *s* of region *r*,*Xs* represent elements of adaptive capacity (indexed by *k*), *Rs* represents indicators for reasons for changes (indexed by *m*), *λ*_*s*_ is a site fixed-effect, *u*_*r*_ is a region fixed-effect, *v*_*c*_ is a binary indicator that captures the effect of crop *c* on adaptation, and *ε* is an idiosyncratic error term.

Model (2) is a count model as the dependent variable (i.e. adaptation intensity) is a count of farming activities. We use a negative binomial regression to estimate the parameters of the adaptation intensity model. The negative binomial regression is a widely used model for count data for its flexibility. It relaxes the assumption of equality in the conditional mean and variance functions imposed by the competing Poisson count model [55, 56]. Specifically, the probability of the count is defined as

$$Prob(Y=y|Z)=\frac{e^{-\lambda }\lambda^{y}}{y!},\;\lambda =e^{Z}$$ (3)

where *Z* is defined as the right-hand side of model (2), specifying dummy variables to account for the site and region fixed effects, *y* is the count measure of adaptation intensity, and exp(*ε*) is a gamma-distributed error term with mean one and variance σ^2^. Note that exp(Z) = exp(*β*X)exp(*ε*), where *β*X is the deterministic part of the RHS of model (2) and includes the elements of adaptive capacity, the stated reasons, and region, site and crop fixed effects. The negative binomial model can be estimated by maximum likelihood, and post-estimation marginal effects (evaluated at the means) are usually calculated for the convenience of interpretation. Refer to Greene [57] for the derivation of the maximum likelihood function and details on the computation of marginal effects.

Our second empirical model is the model of adaptation by diversification. This model regresses the log of the HHI on adaptation intensity, adaptation intensity squared, elements of adaptive capacity, reasons for adaptation, and site, region, and crops fixed-effects:

$${log(HHI)}_{isr}={\alpha_{1}I}_{isr}+{\alpha_{2}I^{2}}_{isr}+\sum_{k}\beta_{k}X_{k,isr}+\sum_{m}\gamma_{m}R_{m,isr}+\lambda_{s}+u_{r}+\sum_{c}v_{c}+\varepsilon_{isr}$$ (4)

We use the logarithm of the HHI to facilitate the interpretations of the parameter estimates as percentage changes. Our construction of the HHI accounts for a very small proportion of zero adaptation values in the data. To incorporate information from these households in the model, we use the log-transformation proposed by McCune and Grace [58] where a very small number is added to each observation in a way that preserves the original order of magnitudes in the data. Mathematically, the log-transformation assumes $\tilde{y}=\ln\left[y+{ln}^{-1}(c)\right]-c$, and *c* = *int*[*ln*(*min*(*x*))], where min(x) is the smallest nonzero value in HHI; int(x) is an integer function that preserves only the integer part of x; y is our non-transformed HHI data, and $\tilde{y}$ is the log-transformed HHI data. In the log-linear model, parameter estimates represent percentage changes in the adaptation diversity index.

In addition to the elements of adaptive capacity, reasons for adaptation, and region, site and crops fixed effects, model (4) includes our measure of adaptation intensity *I* as a regressor. We opted for this specification because the HHI index is affected by the number of farming activities. For instance, if a farmer only changes one activity, his adaptation profile is, by default, not diversified. Moreover, we add the squared term *I*^2^ to investigate whether or not adaptation by diversification has increasing, decreasing, or constant marginal returns to adaptation intensity. This approach allows us to estimate marginal effects of other determinants of diversity while controlling for the default (or expected) behavior of the HHI with respect to intensity. Once we control for this effect in our model, we are able to identify: i) elements that contribute only to intensification; ii) elements that contribute only to diversification; iii) elements that are complementary inputs and increase both the intensity and diversity of adaptation and iv) elements that are substitutes inputs in the sense that they increase diversification but decreases intensification.

Note that the normalized HHI is bounded between 0 and 1, and, as a result, the log-transformed index has upper and lower truncation points. Therefore, the diversification model is estimated with a truncated regression model. This strategy is often applied, for instance, in the technical efficiency literature as efficiency scores, like our diversity index, are truncated between 0 and 1 [59]. Suppose the log of HHI, *y*, follows a normal distribution *N*(*μ*,*σ*^2^), and *y*_*i*_ ∈ (*a*,*b*) has a truncated normal distribution where *a* and *b* are respectively the lower and upper truncation points. The likelihood function for this truncated normal specification is given by:

$$L(\mu ,\sigma ,a,b|y)=\prod_{i=1}^{n}f(y_{i};\;\mu ,\sigma ,a,b)=\prod_{i=1}^{n}\frac{\frac{1}{\sigma }\phi (\frac{y_{i}-\mu }{\sigma })}{\Phi (\frac{b-\mu }{\sigma })-\Phi (\frac{a-\mu }{\sigma })}$$ (5)

where *f*(∙) ≠ 0 is the probability density function for *y*_*i*_ ∈ (*a*,*b*), *ϕ* is the probability density function of a standard normal distribution, Φ is its cumulative distribution function, and *μ* is the deterministic part of model (4).

## Results and discussion

We begin our presentation of results by looking at the variability in average values of our intensification and diversification measures across regions, countries, and sites. We then compare determinants of these two measures in a global model. Finally, we compare global and local level determinants for both measures.

### Summary statistics of intensification and diversification by region and site

Table 2 presents summary statistics for the two adaptation indices used in this study. The results show significant heterogeneity both between and within regions. For instance, households in Central America have the lowest regional level of adaptation intensity with an average of 8.8 practices. This level contrasts with the 11.7 practices in East Africa (the region with highest adaptation intensity), and with the global average of 9.9 practices. Adaptation by diversification is also low in Central America (HHI = 0.219) compared to other regions and the global average (HHI = 0.159). Recall that the normalized HHI takes values from 0 to 1, where 0 represents a household that is perfectly diversified (i.e. an equal share of the farming practices are changed in each activity category), and 1 represents a household that concentrates all adaptive farming practices in one category.

**Table 2:** Descriptive statistics of adaptation intensity and diversification.

Region/Country/Site Code	Adaptation Intensity (activity count)		Adaptation Diversification (HHI index)	
	Mean	Std. Dev.	Mean	Std. Dev.
***West Africa***	***10*.*597***	***5*.*739***	***0*.*167***	***0*.*243***
Burkina Faso–BF01	11.650	6.260	0.265	0.283
Ghana–GH01	15.057	5.243	0.035	0.052
Mali–MA01	5.213	3.458	0.280	0.312
Niger–NI01	8.429	4.424	0.204	0.246
Senegal–SE01	12.703	2.743	0.054	0.074
***East Africa***	***11*.*722***	***7*.*758***	***0*.*165***	***0*.*250***
Ethiopia–ET01	4.143	4.067	0.380	0.384
Kenya–KE01	11.626	4.977	0.111	0.137
– KE02	22.071	5.283	0.026	0.035
Mozambique–MZ01	10.750	4.474	0.116	0.138
– MZ02	4.086	2.673	0.298	0.332
Tanzania–TZ01	17.721	6.729	0.070	0.136
Uganda–UG01	9.836	6.134	0.238	0.290
– UG02	13.543	6.030	0.126	0.167
***South Asia***	***9*.*324***	***5*.*913***	***0*.*144***	***0*.*231***
Bangladesh–BA01	11.421	7.597	0.148	0.306
– BA02	11.207	6.693	0.118	0.229
– BA03	4.443	6.461	0.273	0.348
– BA04	6.114	4.337	0.225	0.309
– BA05	7.479	6.753	0.251	0.354
– BA06	8.364	4.682	0.208	0.337
– BA07	6.329	5.352	0.200	0.305
India–IN08	6.036	5.703	0.154	0.249
– IN09	9.736	4.252	0.110	0.127
– IN10	8.964	5.669	0.068	0.049
– IN11	11.271	4.515	0.092	0.113
– IN12	14.021	7.912	0.040	0.061
– IN13	10.607	5.301	0.096	0.102
– IN14	10.093	5.860	0.096	0.140
– IN16	14.057	5.406	0.052	0.092
– IN17	13.593	5.291	0.043	0.052
Nepal–NE01	7.636	3.603	0.171	0.193
– NE02	8.143	3.136	0.269	0.311
– NE03	9.964	2.457	0.141	0.162
– NE04	9.029	2.767	0.188	0.200
– NE05	7.321	3.539	0.110	0.148
***Central America***	***8*.*816***	***5*.*923***	***0*.*219***	***0*.*281***
Costa Rica–CR04	5.460	4.769	0.280	0.316
Nicaragua–NC01	14.786	4.256	0.076	0.056
– NC02	5.414	4.415	0.364	0.346
– NC03	9.579	4.609	0.184	0.245
**Total Sample**	**9.943**	**6.408**	**0.159**	**0.244**

The table also allows us to compare variation in adaptation levels between sites. We find substantial differences in the adaptation profile of sites within the same region. For instance, while the average adaptation intensity in East Africa is 11.7 activities, we find sites in this region with averages much higher (e.g. 22.1 practices in KE02; 17.7 practices in TZ01), and others with averages are much lower (e.g. 4.1 practices in ET01 and MZ02). Similar patterns can be found in terms of adaptation by diversification. For instance, while the average HHI in West Africa is 0.167, the average HHI of households in the Senegal site (SE01) is 0.054 (almost no specialization, or almost perfect diversification) while the HHI of the Mali site (MA01) is more than four times higher, equal to 0.280.

In summary, we find substantial heterogeneity between regions, countries, and sites with respect to both adaptation intensity and adaptation diversification. Interestingly, in specific regions or sites, less intensified adaptation can be highly diversified, and vice versa. For example, South Asia has the second lowest adaptation intensity on average while it also has the highest adaptation diversification among all four regions. These patterns between adaptation intensification and diversification highlight the importance of understanding adaptation in several dimensions and multiple spatial scales.

### Determinants of intensification and diversification of adaptation at the global level

Results of the global models indicate numerous significant determinants for both intensification and diversification, but there are many more significant determinants for intensification than for diversification (see Table 3). Overall, results indicate that the importance of determinants may differ depending on whether intensification vs. diversification of adaption is considered. Moreover, if a determinant is significant in both models, it may, or may not, cause intensification and diversification to move in the same direction. Note that in the HHI model, a negative sign on a coefficient indicates increasing diversification. The global HHI model indicates that increasing intensification increases diversification at a decreasing rate (see the first two rows in Table 3).

**Table 3:** Determinants of intensification and diversification of adaptation at the global level.

	Adaptation Intensification (activity count)	Adaptation Diversification (HHI index)
Count of adaptation activities		-0.177*** *(0*.*013)*
Count of adaptation activities squared		0.003*** *(0*.*000)*
**ACCESS TO INFORMATION & HUMAN CAPITAL**		
Access to weather information	0.549*** *(0*.*133)*	-0.093* *(0*.*050)*
Membership in farming association(s)	0.370*** *(0*.*109)*	-0.040 *(0*.*035)*
Highest level of education attained is primary	0.320 *(0*.*204)*	0.020 *(0*.*063)*
Highest level of education attained is secondary	0.380* *(0*.*207)*	0.027 *(0*.*065)*
Highest level of education attained is post-secondary	0.461** *(0*.*213)*	0.009 *(0*.*070)*
**FINANCE**		
Access to agricultural credit	0.576*** *(0*.*124)*	-0.019 *(0*.*045)*
Bank account	-0.076 *(0*.*113)*	0.014 *(0*.*044)*
Cash from the government	0.206* *(0*.*110)*	-0.002 *(0*.*043)*
Income from non-farm employment	0.184* *(0*.*108)*	0.088** *(0*.*034)*
Income from renting out land or machinery	-0.092 *(0*.*106)*	-0.077* *(0*.*044)*
**ASSETS**		
Count of household assets	0.028 *(0*.*028)*	0.005 *(0*.*011)*
Livestock	0.583*** *(0*.*152)*	-0.051 *(0*.*051)*
Motorcycle	0.180 *(0*.*120)*	0.012 *(0*.*047)*
Car or truck	-0.163 *(0*.*250)*	0.072 *(0*.*085)*
Boat	0.306 *(0*.*516)*	-0.343** *(0*.*147)*
**FARM & HOUSEHOLD CHARACTERISTICS**		
Running water	0.223 *(0*.*516)*	-0.061 *(0*.*064)*
Storage facility for crops	0.545*** *(0*.*143)*	-0.062 *(0*.*057)*
Planted trees	0.260*** *(0*.*118)*	-0.004 *(0*.*028)*
Number of individuals in a household	0.002 *(0*.*098)*	-0.003 *(0*.*003)*
Household is female-headed	-0.373** *(0*.*008)*	0.072 *(0*.*054)*
**FARMING & CRISIS EXPERIENCE**		
Farming experience is at least 10 years	1.610*** *(0*.*219)*	-0.066 *(0*.*061)*
Experienced climate crisis in the last 5 years	-0.177 *(0*.*158)*	-0.024 *(0*.*063)*
**STATED REASONS FOR CHANGES**		
Market conditions	5.152*** *(0*.*266)*	-0.013 *(0*.*060)*
Climate variability	0.923*** *(0*.*145)*	0.008 *(0*.*037)*
Pests and disease	0.681*** *(0*.*117)*	0.052 *(0*.*043)*
Government/NGO intervention	0.376** *(0*.*160)*	-0.167*** *(0*.*054)*
Labor availability	0.978*** *(0*.*120)*	0.141*** *(0*.*047)*
Land productivity	0.855*** *(0*.*118)*	-0.113*** *(0*.*043)*
**N**	**5,280**	**4,343**

The table reports estimates of marginal effects for the adaptation intensification count model. Estimates of the adaptation diversification model represent percentage changes in the HHI index. Both regressions include region, site, and crop fixed effects, estimated by the inclusion of dummy variables. These estimates are provided in S3 Table. Cluster-robust standard errors at the village level are *in italics* and in parentheses.

Significance levels are

* p<0.1

** p<0.05

*** p<0.01.

We also estimated a diversification model with linear intensity and results are very similar to the ones reported here.

In general, the explanatory variables for access to information and human capital for the count model are statistically significant drivers for intensification of adaptation practices, with the exception of primary education. These indicators increase the number of activities undertaken by approximately 1/3 to 1/2 of an activity. In contrast, for the diversification model, only access to weather information is statistically significant, and increases diversification of adaptation practices, on average, by 9%. Surprisingly, while secondary and post-secondary educations are significant determinants of global intensification, we do not find statistical evidence of their effect on global diversification. Overall, though many types of access to information and human capital are significant parts of household capacity to increase the intensity of adaptation, only access to weather information also promotes diversification.

Moser and Barrett [60] identified financial constraints as an important barrier to the use of modern agricultural inputs such as fertilizers. A recent study [31] finds a similar result in the context of adaptation to climate change. Three out of five explanatory variables for finance in our count model are statistically significant drivers for intensification of adaptation practices. Having access to agricultural credit, receiving cash from government, and earning income from non-farm employment increase the number of activities changed by approximately 1/5^th^ to ½ of an activity. For the diversification model, earning income, either from non-farm employment or from renting out land or machinery, is statistically significant. However, these two drivers have contrary influences on adaptation diversification. While non-farm employment decreases adaptive diversification by approximately 9%, income from land or machinery rentals increase adaptive diversification by approximately 8%. It appears as though households that are diversified away from agricultural production with off-farm jobs have less of a need to pursue adaptive diversification than those pursuing agriculturally-related income through land and machinery rentals.

With respect to assets, we find, somewhat surprisingly, that only livestock ownership positively impacts adaptation intensity, which, on average increases approximately 0.6 activities. In contrast, the only asset variable that influences adaptation diversification is boat ownership. Households that own a boat (only 0.8% of the total sample–see S2 Table), are approximately 34% more diversified than those who don’t; the largest determinant of adaptation by diversification.

For farm and household characteristics in the count model, the presence of physical and natural capital is shown to increase adaptive activities. Storage facilities for crops and planted trees positively influence adaptive intensity, causing increases of approximately ½ and ¼ of an activity, respectively. In contrast, female-headed households are negatively associated with numbers of adaptive activities, causing decreases of approximately 1/3 of an activity. We do not find statistical evidence of significant effects of farm and household characteristics on diversification.

Of the two variables specified in our models to capture farming and climate crisis experience, only farming experience (of at least 10 years) is statistically significant, and positively related to adaptive intensification. Households having this experience tend to, on average, undertake 1.6 more activities. This result indicates that farming experience is the most important element of adaptive capacity, influencing intensity, in our analysis.

In addition to variables that could affect the adaptive capacity of households, we also have data on reasons why households made changes. In the model of adaptation by intensification, all reasons are positive and significant, with impacts ranging from increases of approximately 1/3 of an activity (for government/NGO intervention) to over 5 activities for market conditions. Note that climate variability is a far smaller influence than markets, increasing adaptive intensification by approximately 1 activity. There are three stated reasons for changes that significantly affect both adaptations by intensification and by diversification. Governments and NGOs interventions lead to increased intensification by 1/3 of an activity, and they are shown to have large effects on adaptive diversification, increasing diversification by approximately 17%. Fluctuations in land productivity also induces synergies as it increases adaptation intensification by approximately 0.9 activities, and increases diversification by approximately 11%. Interestingly, households in our global sample tend to increase intensification by 1 activity due to labor availability. Instability in labor supply, however, induces households to increase adaptive specialization by 14%.

Though the fixed effects in the models are mainly included as control variables, they nonetheless disclose some interesting findings (S3 Table). For example, all of the crop fixed effects in the global models, except millet, are positive and significant with respect to adaptive intensification (relative to the base case of all other not listed sample crops). That is, households cultivating these listed crops usually adopt more activities than cultivating other crops. Some of the larger adaptive intensification activities are associated with rice (including its variety rice-Aman) and wheat production. There are far fewer significant effects with respect to the diversification global model, but maize, beans, mustard, banana, potatoes and coffee are all associated with increased adaptive diversification while no evidence is found for other crops.

The estimation of global crop fixed effects suggests that six crops cause adaptation with respect to both intensification and diversification. These crops are maize, beans, mustard, banana, potatoes and coffee. Maize, for instance, causes farmers to adopt an additional 1.5 farming practice and to increase diversification by 19% (when compared to baseline crops). These finding agree with the current literature on the incremental and transformative adaptation needs for these crops. Transformative adaptation will be required for 30% of current maize and banana areas and for 60% of bean areas in Africa [61], while globally areas to cultivate coffee will decrease by 50% by 2050 [62].

### Adaptation at global and local scales

The CCAFS dataset used in this study is composed of 5,314 households, with approximately 140 household interviews for each of 38 sites [41, 42]. This survey design offers a rare opportunity for a comparative investigation regarding the key determinants and relationships between intensification and diversification *across scales*. To the best of our knowledge, this study is the most geographically comprehensive examination of agricultural adaptation of smallholder farmers in developing countries, and the first to report results across scales around the globe.

We use site-level data to estimate determinants of both measures of adaptation (intensification and diversification) for each site. Site-level results for intensive and diverse adaptation are summarized, respectively, in Table 4 and Table 5. Many determinants are shown to have insignificant impacts on intensive adaptation, despite their overall significance at the global level. These differences could be a reflection of differing local conditions, and/or a result of lower statistical power due to the smaller sample size. But in some cases, results at the local level are significant where as they are not at the global level. For example, recall that our global models identify numerous significant global determinants of adaptation intensity, while only a few significant global determinants of diversification. But somewhat surprisingly, despite the insignificance of many potential determinants in the diversification global model, many of the site level determinants are significant. These differences in results suggest that homogeneity of impact at the site level, overtakes the greater statistical power at the global level. Overall, the results suggest that while adaptation by intensification can be influenced by large scale policies, the determinants of adaptation by diversification vary significantly with geographical boundaries and require localized and targeted policy interventions. We explore these results in more detail in the paragraphs that follow.

**Table 4:** Effects of elements of adaptive capacity and stated reasons on *adaptation intensification* at the site level.

	West Africa					East Africa								South Asia																					Central America			
	BF	GH	MA	NI	SE	ET	KE		MZ		TZ	UG		BA							IN									NE					CR	NC		
	01	01	01^a^	01	01	01^a^	01	02	01	02	01	01	02	01^a^	02	03	04	05	06	07	08	09	10	11^a^	12	13	14	16	17	01	02	03	04	05^a^	04^a^	01^a^	02	03
**ACCESS TO INFORMATION & HUMAN CAPITAL**																																						
Access to weather information	O	O	O	O	O	O	O	O	O	-	O	O	O	O	+	+	+	O	O		O	O	+	-	+	+	O	+	O	O	O	+	O	O	O	-	+	O
Membership in farming association(s)	+	-	O	O	O	+	O	O	+	O	+	O	O	+	O	O	O	+	O	O	O	+	O	O	O	+	O	O	O	O	+	O	O	O	+	+	O	O
Highest level of education attained is primary	O	O	O	O	O	O	O	O	O	O	+	O	+	O	+	-	O	+	O	O	O	O	O	O	+	-	-	O	O	O	O	+	O	O	+	+	O	O
Highest level of education attained is secondary	O	O	O	O	-	O	O	O	O	+	O	O	+	+	+	O	O	+	O	O	O	O	O	O	O	-	O	O	O	O	O	+	O	O	+	O	O	O
Highest level of education attained is post-secondary	O	O			-	O	O	O		+	O	O	+	+	+	O	O	O	O	O	O	O	O	+	O	-	-	O	O	-	O	+	O	O	+	+	O	O
**FINANCE**																																						
Access to agricultural credit	+	+	O	+	O	-	O	+	+		O	O	O	O	O		+	+	-	+	O	O	+	O	O	O	O	O	O	O	+	O	+		+	O	O	O
Bank account	-	O	O		O	O	O	O	O	O	O	O	-	O	O	O	O	O	-	+	+	O	O	O	O	-	O	+	O	O	O	O	O	O	O	O	+	O
Cash from the government	O	O	O	O	+	O	O	O	O	O	O	+	O	O	O	+	+	O	O	-	O	O	O		-	+	O	O	O	O	O	O	O	O	+	O	O	O
Income from non-farm employment	+	O	O	-	O	+	O	O	O	+	O	O	O	O	O	O	O	-	O	O	O	O	O	O	O	+	O	-	O	O	O	O	O	O	O	O	O	O
Income from renting out land or machinery	O	+	O	O	+	O	O	O	O	O		O	O	O	O	O	O	O	O	O	O	-	O	O	O	O	O	+	O	O	O	+	O	O	+	O	O	O
**ASSETS**																																						
Count of household assets	O	O	O	O	O	O	O	+	O	O	+	O	O	+	O	O	O	O	O	+	O	O	O	O	O	O	O	O	O	+	+	O	O	O	O	+	O	O
Livestock	O	+	O	O		O	O	+	O	O	O	O	O	O	+	O	O	-	O	-	+	O	O	+	O	O	O	O	O	+	O	O	-	O	+	O	O	O
Motorcycle	O	+	O	O	O		O	-	O	-	+	O	O	O	+	O		+	O	-	O	O	O	O	O	O	O	+	O	O	O	-	O	-	O	O	O	O
Car or truck			O		O		O	-	O	+	-	-	+	+	O	-		O			O	O	O	-	O	O	+	O	O			O		O	O	-	O	+
Boat		+							O				-	+	-	O	O	O	+	+					-					+								
**FARM & HOUSEHOLD CHARACTERISTICS**																																						
Running water		+			+		O	O	O	O	O	O	O	O	+	O	O	O	O	-	O	O	O		O	O	-	O	O	O	+	+		O	O	O	O	-
Storage facility for crops	O	+	O	O	O	O	O	+	O	-	O	O	O	O	O	+	+	O	O	+	-	O	O	+	O	O	O	O	O	O	+	+	+	O	O	O	+	O
Planted trees	+	O	O	+	+		O	+	O	O	O	+	O	O	O	O	O	O	O	-	O	O	O	O	O	O	O	-	O	O	O	+	O	O	+	+	O	+
Number of individuals in a household	O	O	O	O	O	-	O	O	O	O	O	O	O	O	O	O	+	O	O	O	O	-	O	O	O	O	O	O	O	O	-	O	O	+	O	O	+	O
Household is female-headed	-	O	O	O	O	O	-	O	O	O	-	O	O	O		O	-	O	O	O	-			O	O	O	-	-	O	+	-	O	O	-	O	O	O	O
**FARMING & CRISIS EXPERIENCE**																																						
Farming experience is at least 10 years	O		O		+	+	O	+	+	O	O	O	O	O	+	+	+	+	+	+	O	-	O	+	O	O	+	-	O	O	O	O	O	O	+	O	+	O
Experienced climate crisis in the last 5 years	O	O	O	-	O	+	O	O	-	-	O	O	-		-						+	O		O	O		O	O	O	O	+	+	O	O	+	O	O	O
**STATED REASONS**																																						
Market conditions	+	+	+	+	+	+	+	+	+	+	O	+	+	+	+	+	+	+	+	+	+	+	+	+	+	O	+	+	O	+	+	+	+	+	+	+	+	+
Climate variability	O	+	+	O	O	+	+		+	O	+	+	O	O	O	+	O	+	+	O	O	-	O	O	O	O	+	O	O	O	O	+	O	-	+	O	+	+
Pests and disease	+	O	+	+	+	O	+	+	+	-	O	O	O	+	O	O	+	O	+	O	O	O	O	O	+	O	O	+	O	O	O	O	O	O	+	O	+	O
Government/NGO intervention	+	+	O	+	+	+	O	-	+	O	O	O	+		+			O			O		-	O	+	-	+		O	+	O	-	O	O	O	+	+	O
Labor availability	O	O	+	+	O	+	O	+	O	O	O	O	+	+	O	+	O	+	O	-	O	O	O	O	O	O	O	-	O	+	+	+	+	+	+	O	+	O
Land productivity	O		+	O	O	O	+	+	O	+	+	O	O	+	+	O	+	+	+	+	O	O	O	O	+	O	O	+	O	O	+	+	+	+	O	O	+	+
**CROP FIXED EFFECTS**																																						
Cowpeas	+								+	O																												
Millet	+		O	+																																		
Sorghum	+	O	+				+																															
Maize		O			O	O				+	+		O											+	+		+	+							+	O	+	+
Peanuts		+			O																																	
Beans				+		+	+				+	+	O								+	+														O	+	O
Leafy Vegetables				O																																		
Groundnuts									+																													
Finger Millet										O																												
Banana											O		+	+																								
Cassava												+																										
Sweet Potato												O																										
Papaya															+															O								
Rice														+	+	O	O	O			+	+	+	+	+	+	+			+	+		+	+				+
Rice–Aman														O	+	+	+	+	+	+																		
Garlic																+		+																				
Coconut																	O			O																		
Lentils																			O																			
Rice—HYV Boro																			O																			
Betel Leaf																				+																		
Wheat						O															+			O	+		+	+		+	+	O	+					
Mustard																							+			+					+	+	+	+				
Potatoes																												+										
Fodder Crop																													O									
Cocoa																																					O	
Sesame			+																																			
Frijol Negro																																			+			
Frijol Rojo																																			-	+		

The two-letter codes in the second row represent country names as specified in Table 2 and Table 3. The numbers in the third row represent site codes consistent with those same tables.

"+" cells indicate positive significant marginal effects at the 10% significance level.

"-" cells indicate negative significant marginal effects at the 10% significance level.

"O" indicates not significant; blank cells indicate omitted variables.

For each site, models include dummy variables for the three most frequently observed crops where adaptation activities were applied to.

^a^ Negative binomial regression model did not converge (OLS regression model with robust standard errors was used).

**Table 5:** Effects of elements of adaptive capacity and stated reasons on *adaptation diversification* at the site level.

	West Africa					East Africa								South Asia																					Central America			
	BF	GH	MA	NI	SE	ET	KE		MZ		TZ	UG		BA							IN									NE					CR	NC		
	01	01	01	01	01	01	01	02	01	02	01	01	02	01	02	03	04	05	06	07	08	09	10	11	12	13	14	16	17	01	02	03	04	05	04	01	02	03
Count of adaptation activities	-	-	-	-	-	-	O	O	-	-	O	O	-	-	-	-	-	-	-	O	O	O	O	O	-	-	O	O	-	-	-	O	O	-	-	+	-	-
Square count of adaptation activities	O	O	O	O	+	+	O	O	+	+	O	O	O	+	+	O	+	O	O	O	O	O	O	O	+	+	O	O	+	O	O	O	O	+	+	-	+	O
**ACCESS TO INFORMATION & HUMAN CAPITAL**																																						
Access to weather information	O	O	O	O	O	O	O	O	O	O	O	O	O		O	O	O	O	O		O	-	+	-	O	+	-	O	O	+	O	O	O	O	O	O	+	-
Membership in farming association(s)	O	O	O	O	O	O	O	O	O	+	O	O	O	+	O	+	O	-	O	O	O	+	O	-	O	O	O	O	O	O	O	O	O	O	O	+	-	O
Highest level of education attained is primary	O	O	O	O	+	+	O	+	O	O	+	O	O	O	O	O	O	-	+	O	O	-	O	O	O	O	+	-	O	+	-	O	O	O	O	O	O	O
Highest level of education attained is secondary	+	O	-	O	O	O	O	O	O	O	+	O	O	O	O	O	O	-	O	O	O	O	O	O	O	O	O	-	O	O	-	+	O	O	O	O	O	O
Highest level of education attained is post-secondary	+	O			O	O	O	O		O	O	O	O	O	O	O	O	-		O	-	-	O	O	-	O	O	-		O	-	O	O	O		-	O	O
**FINANCE**																																						
Access to agricultural credit	O	O	O	O	-	-	O	-	-		+	O	O	O	O		O	O	O	O	-	O	O	O	+	O	O	O	+	O	+	O	+		-	O	O	O
Bank account	O	-	O		O	+	O	O	O	-	O	O	O	O	O	O	+	O	O	O	O	O	-	O	O	-	O	O	O	O	+	+	O	O	O	O	O	-
Cash from the government	O	O	O	O	O	-	O	O	O	O	+	-	O	O	O	-	O	O	O	+	O	O	+		-	O	O	+	O	-	O	O	O	O	O	O	O	+
Income from non-farm employment	+	O	O	+	O	-	O	O	O	O	O	+	O	O	O	O	O	O	O	O	O	O	O	O	O	+	O	+	O	O	O	O	O	O	O	+	-	O
Income from renting out land or machinery	O	O	-	O	O		O	O	O	O		O	O	O	-	O	O	O	O	O	O	+	O	O	-	O	O	+	-	O	+	O	O	O	O	O	O	-
**ASSETS**																																						
Count of household assets	O	O	O	O	O	O	O	O	-	O	O	O	O	-	O	O	O	O	O	-	O	O	O	O	O	O	O	O	O	O	O	O	O	O	O	-	O	O
Livestock	O	O	O	+			O	O	O	-	-	O	O	O	O	O	O	O	-	O	O	O	O	O	-	O	O	O	O	O	-	-	-	+	O	O	O	O
Motorcycle	O	O	O	+	O		O	O	O	O	O	O	O	O	O	O		O	O	+	O	O	-	O	+	O	O	O	O	O	O	O	O	O	O	+	O	O
Car or truck			O		O		-	O	O	O	O	-	O	+	O			O			-	O	+	+	+	+	O	O	O			-		-	O	+	O	+
Boat		+							O				O	O	-	O	-	-	O	-					O					O								
**FARM & HOUSEHOLD CHARACTERISTICS**																																						
Running water		O			O		-	O	O	+	O	O	O	-	O	O	O	O	O	O	O	O	-		O	+	+	O	-	O	+	O		+	-	O	O	O
Storage facility for crops	O	O	-	O	O	O	O	O	O	O	O	O	-	+	O	+	-	O	O	O	O	O	+	-	O	O	O	O	O	O	-	O	O	-	O	O	O	O
Planted trees	O	O	O	O	O		O	O	O	+	O	O	O	O	O	O	O	O	O	O	O	O	O	O	O	+	O	O	O	O	O	O	O	O	O	O	O	O
Number of individuals in a household	O	O	O	O	O	O	O	O	O	O	O	O	O	O	+	O	O	O	O	O	+	O	+	O	O	-	O	O	O	O	O	0	O	O	+	O	O	O
Household is female-headed	O	O	O	O	-	O	O	O	O	O	O	+	O	O				O	O	O				-	O	O	O	O	O	O	O	O	O	O	O	O	O	O
**FARMING & CRISIS EXPERIENCE**																																						
Farming experience is at least 10 years	O				+	O	O	O	O	O	O	O	O		O		O	O		+	O	O	O	O	O	O	-	O	O	O	+	O	O		O		O	O
Experienced climate crisis in the last 5 years	O	O	+	-	O	O	O	O	O	+	+	-	+		+						O	O		-	O		O	O	O	O	O	O	+	O	-	O	-	O
**STATED REASONS**																																						
Market conditions	O	O	O	+	-	-	+	O	+	O	O	O	O	O	O	+	+	O	O	O	O	O		O	O	-	-			-	O	-	O	-	O		O	O
Climate variability	O	O	O	O	O	O	O		O	O	O	O	O	O	+	O	O	O	O	O	-	-	O	-	O	O	-	O	+	O	-	O	O	-	O	O	O	O
Pests and disease	O	O	-	+	O	-	O	O	O	O	O	O	O	O	O	O	O	+	O	O	O	-	O	+	+	O	+	O	O	-	O	-	O	+	+	-	O	-
Government/NGO intervention	O	O	-	O	O	-	O	O	-	O	O	O	O		O			O			O		+	O	O	+	O		O	O	O	O	O	+	O	O	-	+
Labor availability	O	O	O	O	O	O	O	O	+	O	O	O	O	O	+	O	O	O	O	O	O	O	O	O	-	O	O	+	-	O	+	+	+	O	O	+	O	O
Land productivity	O		O	-	O	O	O	O	O	+	O	O	O	O	O	O	O	O	O	O	O	O	+	+	+	O	O	-	O	O	O	O	-	O	O	O	O	O
**CROP FIXED EFFECTS**																																						
Cowpeas	-								-	O																												
Millet	O		O	-																																		
Sorghum	O	O	O				O																															
Maize		-			O					O			-											-	O		-	O							O	-	O	O
Peanuts					-																																	
Beans				O		O	-				O	O	+								-	O														-	O	O
Leafy Vegetables				O																																		
Groundnuts																																						
Finger Millet										O																												
Banana											O		-	O																								
Cassava												O																										
Sweet Potato												O																										
Papaya															O															O								
Rice														+	O	O	O	O						-	O		O			O	O		O					O
Rice–Aman														O	O	O	O	O	+	-																		
Garlic																O		O																				
Coconut																	+			O																		
Lentils																			+																			
Rice—HYV Boro																			O																			
Betel Leaf																				-																		
Wheat						O															O				O		O	+		O	+	-	O					
Mustard																							-			-					O	O	+	O				
Potatoes																												O										
Fodder Crop																													O									
Cocoa																																					O	
Sesame			O																																			
Frijol Negro																																			O			
Frijol Rojo																																			O	-		

The two-letter codes in the second row represent country names as specified in Table 2 and Table 3. The numbers in the third row represent site codes consistent with those same tables.

"+" cells indicate positive significant marginal effects at the 10% significance level.

"-" cells indicate negative significant marginal effects at the 10% significance level.

"O" indicates not significant; blank cells indicate omitted variables.

For each site, models include dummy variables for the three most frequently observed crops where adaptation activities were applied to.

For the adaptation diversification local models, as in our global approach, we include the count of farming practices changed as a determinant of the HHI index (first two rows of Table 5). Following the global results, the site diversification models indicate that, overall, increasing intensification increases diversification at a slightly decreasing rate.

#### Access to information and human capital

The global results, that information and human capital positively affect adaptation intensification, tend to hold for some sites; one site in Uganda, two sites in Bangladesh, the site in Costa Rica and one site in Nicaragua. Surprisingly, there are three sites (one in Senegal and two in India) where education tends to decrease the number of adaptive activities undertaken. Access to weather information helps improve adaptation intensification in many South Asia sites, while little similar evidence is found for other regions. India has allocated government resources to producing and distributing weather forecasts for the agricultural sector (http://www.tropmet.res.in/). Perhaps because of these efforts, many of our results for study sites in India indicate that access to weather information has had a positive impact on increasing the intensity of adaptation. Another study similarly [63] found that agro-weather information enhances the adoption of climate smart agricultural practices in Kenya. However, the author also points out that access remains low among smallholder farmers.

Membership in farming associations is particularly prevalent in influencing increased numbers of adaptation activities across several sites. Along these lines other studies [64, 65] found that membership in farmers’ organization in Nigeria were positively correlated with the adoption of new practices. Though our Nigerian site did not show similar results in terms of adaptation intensity, our findings at several African sites are consistent with these two studies.

The significant impact of access to weather information on adaptation diversification at the global level (Table 3) is only evident at three sites in India and one site in Nicaragua (Table 5). But there are also several sites where access to weather information, instead, significantly increases specialization (i.e. decreases diversification), including two sites in India, one site in Nepal and one site in Nicaragua. There are also some other variables related to access to information and human capital that significantly influence diversification/specialization at the site level. For example, attaining a primary level of education increases diversification of adaptation in four sites in South Asia, but can also increase specialization at some sites in this region, as well as in West and East Africa. Moreover, though post-secondary education increases diversification at several sites in Bangladesh, India, Nepal and Nicaragua, it increases specialization of adaptation activities at the site in Burkina Faso. In general, we find evidence that access to information and human capital increase diversification at some sites in South Asia and Central America and increase specialization in West and East Africa. The positive effect of climate and weather information is recognized by a comprehensive study conducted by the World Meteorological Organisation (WMO) stating that the Benefit Cost Ratio (BCR) of National Meteorological and Hydrological Services in developing countries range from 4 to 1 to 36 to 1 [66].

#### Finance

Recall that our global model results indicate that three financial variables (credit, cash from government, and non-farm employment) positively affect adaptation intensity. These intensification effects are reflected at a number of sites for two variables; access to agricultural credit and cash from the government. Nevertheless, our site level analysis reveals significant heterogeneity. While our findings suggest that local government policies that involve cash transfers induce adaptation by intensification in sites in Senegal, Uganda, Bangladesh, India, and Costa Rica, many other sites in Africa, South Asia, and Central America, do not have such effects. In general, access to agricultural credit has a positive effect on the adaptation intensity of households in several African countries, Bangladesh, Nepal, and Costa Rica, while our results indicate that credit markets in India and Nicaragua do not encourage adaptation intensity. Income from the other finance variables (i.e. non-farm employment, renting out land or machinery, or having a bank account) is significant at fewer sites, and may increase or decrease adaptive intensification.

The site level diversification models reveal a variety of effects of financial determinants. For non-farm income, there are impacts of increased adaptive specialization at six sites, while adaptive diversification is only impacted at two sites. For land and machinery rental income, there are impacts of increased adaptive diversification at five sites, while adaptive specialization is only impacted at three sites. Three explanatory variables (i.e. access to agricultural credit, bank account, and cash from the government) are shown to be insignificant in the global diversification model, but are significant at several sites. Access to agricultural credit drives diversification in six sites (four are in Africa) and specialization in five sites (four are in South Asia), while having a bank account causes diversification in five sites and specialization in four sites (three in South Asia). Finally, receiving cash from the government has significant impacts at 10 sites (six in South Asia), with results split between driving diversification or specialization.

#### Assets

Globally, livestock was the only asset in our sample that was identified as a statistically significant determinant of adaptation intensity. Comparing this result to those from the site models, livestock is shown to increase adaptive intensity at 7 sites, while it decreases counts at 3 sites. There are many sites where livestock is shown to not influence adaptive intensity. For example, the site in Ethiopia is mainly a pastoral site, so there are fewer crops cultivated. There are also a number of explanatory variables that, while not significant at the global level, are significant drivers of adaptive intensification at the site level. For example, the count of household assets enhances adaptive intensification across seven sites (in Kenya, Uganda, Bangladesh, Nepal, and Nicaragua). There are also influences of owning various forms of transportation, with mixed results across regions and sites. At some sites in Ghana, Tanzania, Bangladesh and India, owning a motorcycle increases intensification of adaptation, while in Kenya, Mozambique, Bangladesh and Nepal, there are sites where a motorcycle decreases adaptive intensification. Owning a car or a truck yields mixed results. In Nicaragua, for instance, there is a site where ownership negatively influences and influences intensification, and another site where the effect is positive. Similarly, in East Africa, car or truck ownership positively influences adaptive intensity at two sites, but has negative effects at three sites. Boat ownership also has the potential to effect adaptive intensification at the site level both positively (at 5 sites) and negatively (at 3 sites), especially for sites close to water bodies, such as those in Bangladesh.

For site level determinants of adaptive diversification, the impact of boat ownership is significant at five sites; four with increased diversification in Bangladesh and one with increase specialization in Ghana. But there are a number of variables that were not significant in the global model that appear significant at a number of sites. Counts of household assets and possessing livestock are generally shown to increase adaptive diversification with significant results on 11 sites. Only two sites show significant impacts on increased adaptive specialization. In contrast, vehicular ownership (i.e. motorcycles, cars or trucks) are shown to have mixed results with 11 sites showing impacts on adaptive specialization, while six sites show impacts on adaptive diversification.

#### Farm and household characteristics

The positive influence of physical and natural capital in promoting adaptive intensity at the global level is largely reflected at the site level. Considering all of these variables together (i.e. running water, storage facility for crops and planted trees), they have a positive effect on 24 sites, and a negative effect on seven sites. Notably, most of these negative effects occur in South Asia (five sites). Confirming the global trend, the negative effect of female-headed households on adaptive intensification also holds for nine sites across regions. A recent study [67] explains that women can be less adaptive because of financial or resource constraints. Males tend to receive information and extension services, and adaptation strategies tend to increase work load for women.

Physical and natural capital variables, which were insignificant in influencing adaptive diversification at the global level, are also significant determinants at a number of sites, but with mixed results. Running water causes increased adaptive specialization on five sites, and also increased adaptive diversification on five other sites. Storage facilities increase adaptive diversification at six sites; however, they are significant determinants of increased specialization at three sites. Planted trees are only significant at two sites, and are shown to cause increased specialization. Though there is no evidence to show that household size is a significant driver of diversification at the global level, four sites (mostly in South Asia), indicate that household size negatively affects diversification. For female-headed households, only three sites indicate significant influences on diversification, with two sites (in Senegal and India) having positive impacts on adaptive diversification and another one (in Uganda) having a negative impact.

#### Farming and crisis experience

Similar to global results, having farming experience of at least 10 years tends to increase adaptive intensity at 14 sites. While having experienced a climate crisis in the last 5 years is not significant in the global model, it does have significant impacts on adaptive intensification at 10 sites, with equal numbers of sites where a crisis increased and decreased adaptive intensity. It appears as though in some cases, a crisis can increase incentives to intensify adaptation, while in other cases, crises may erode adaptive capacity and lead to reduced adaptive intensification. We find that the erosive effect is more common in Africa (especially East Africa), whereas positive intensification effects are observed in India, Nepal, and Costa Rica.

We also find some significant impacts of farming and crisis experience in influencing adaptive diversification at the site level. There are three sites where adaptive specialization is increased by farming experience and one site where this variable increases adaptive diversification. Climate crisis is a more common site level determinant of diversification than is farming experience. The effects again depend on the region and are mixed. For households that have experienced crises, six sites have significant impacts on adaptive specialization, and five sites have significant impacts on adaptive diversification.

#### Stated reasons for changes

Site level intensification results follow patterns that are similar to global effects. Market conditions are shown to be the main motivator of adaptive intensification and are significant determinants of adaptation in all but three sites. The importance of markets is widely observed, perhaps because markets offer incentives to increase production or quality by adopting new or changing existing practices. Moreover, markets can provide financial resources to support investments in adaptation.

Climate variability is also an important driver of adaptation intensification in several sites, but not Indian sites. It is not clear why climate variability in India is not a key driver for adoption of practices but may be related to the subsidized weather insurance schemes that are being provided by the government to all farmers (Indian Weather Based Crop Insurance Scheme). For other countries, it is evident that climate variability can be an important factors that causes farmers to adapt.

Most of the other reasons have significant impacts on increasing adaptive intensification on approximately half of the sites, but with significant variation between countries. For example, while our results indicate that labor availability and land productivity are important determinants of adaptation intensification on almost all sites in Nepal, these determinants are not significant on most sites in India.

There are far fewer significant reasons for influencing adaptive diversification at the site level. For the three variables that were significant in influencing adaptive diversification at the global level (i.e. Governments/NGO interventions, labor availability and land productivity) there are a number of significant impacts, with mixed results. For the eight sites where Government/NGO interventions are significant, four increased adaptive diversification, while four increased adaptive specialization. Similarly, for the seven sites where land productivity was a significant reason for adaptation, three increased adaptive diversification while four increased adaptive specialization. An exception to these mixed results occurs for labor availability, where this variable promoted adaptive specialization on seven of the nine sites where this variable was significant.

#### Crop effects

At the site level, the three most important crops reported by surveyed households in each site are included in our models to control for the effects of crop mixes on adaptation. For the site level intensification models, almost all significantly estimated marginal effects are positive, indicating more intensified adaptation on these selected crops than “others” which was the baseline category. Cultivating Frijol Rojo in Costa Rica is the only exception where a lower level of intensification is found. In contrast to the consistent positive effect across sites in the intensification model, there is more variability associated with site level crop effects and adaptive diversification, especially for several sites in South Asia. In these sites, there are some cultivated crops that lead to more diversified adaptation, while others lead to more specialized adaptation.

## Conclusions

### Global drivers of adaptation

The results of the global models of adaptation reveal central tendencies that are significant across rural areas of 15 developing countries. We classify elements of adaptive capacity in five categories (information and human capital, finance, physical assets, farm characteristics, and experience) and find statistically significant global determinants of adaptive intensification in each of these categories. Most variables in these categories are significant, except for the asset variables where only livestock is found to increase intensive adaptation. For adaptive diversification, we find significant global effects in four categories (all but experience). Interestingly, the diversification adaptation model shows only five statistically significant drivers (in contrast with 14 for intensification), and one of these, income from non-farm employment, significantly increases specialization. Governments wishing to diversify adaptive responses could provide access to weather information. Moreover, those households that receive income from renting out land or machinery or own a boat are likely to be undertaking more diversified adaptation.

We also investigate reasons for change at the global level. The main reason for adaptation by intensification is the market, which is responsible for approximately 5 practices changes. For diversification, the main driver is government and NGO intervention, leading to 17% increases in the diversification index. Results also indicate that climate variability is an important reason for adaptation intensity and leads to approximately 1 additional farming practice changed. However, climate variability is one of several reasons, including labor availability and land productivity, that is having similar effects on adaptation intensity; all of which are well below the impacts of market conditions. Moreover, climate is not a driver of adaptation diversity. These results suggest the importance of understanding adaption in response to multiple changes beyond climate change.

### Patterns of adaptation across scales

We compare our global results to those obtained by site-level empirical models. Many of the global results are consistent with results at the site level. But there are also numerous cases where globally significant determinants are not generally reflected on individual sites, and where individual sites show significant determinants of adaptation that are not reflected as global effects.

Overall, results indicate substantial variation in the dynamics of local agriculture, both within and between regions. For instance, with respect to intensification adaptation, we find that access to weather information does not seem to affect the number of activities undertaken in Africa, but is an important determinant in several sites in Bangladesh and India. Likewise, with respect to diversification adaptation, owning a car or truck tends to increase adaption diversification on several sites in India, but has the opposite effect of increasing specialization at two sites in Nepal.

This manuscript identifies variations in the determinants of adaptation across scales. Behind these variations are numerous stories about why adaptation occurs, as it does, at specific sites. We hope that this paper induces more comparisons between local and global conditions, which could ultimately improve our understanding of drivers of different types of adaptation and their relevant scales. We recognize that conclusions based on local vs global data is empirically challenging, as sample sizes significantly vary between the two types of analyses. Making conclusions based on tests with varying statistical power is a limitation of this research. This limitation makes it even more important to understand the underlying mechanisms that result in local vs global decoupling found in the paper.

### Policy implications

Results indicate that the two dimensions of adaptation that we investigate (i.e. intensity and diversification) are influenced by different sets of drivers. Results at the global scale indicate that, while there are numerous avenues through which policy intervention can favour intensification, there are limited options for influencing diversification. Despite these differences, we find a similarity between drivers of intensification and diversification adaptation. Our global model results indicate a synergistic element of adaptive capacity that promotes both intensification and diversification: access to weather information.

Our global models identify numerous significant global determinants of adaptation intensity, while only a few significant global determinants of diversification. But somewhat surprisingly, despite the lack of significance of many potential determinants in the diversification global model, many of the site level determinants are significant. These differences in results suggest that the greater homogeneity of impact of drivers on diversification adaptation at the site (as opposed to the global) level, overtakes the greater statistical power due to a larger number of observations at the global level. Overall, the results suggest that while adaptation by intensification can potentially be influenced by large scale policies, the determinants of adaptation by diversification vary significantly with geographical boundaries and require localized and targeted policy interventions.

Despite our findings of more homogeneity in intensity adaptation than diversification adaption across sites, we find substantial heterogeneity between regions, countries, and sites across both dimensions. In specific regions or sites, less intensified adaptation can be highly diversified, and vice versa. For example, South Asia has, on average, the second lowest adaptation intensity, yet it also has the highest adaptation diversification among all four regions. These varying patterns between adaptation intensification and diversification highlight the importance of understanding adaptation in several dimensions and at multiple spatial scales.

Our findings also indicate the importance of considering adaptation in the context of many different types of driving circumstances. Though climate change has arguably been a large stimulus for research into adaptation, our results regarding reasons for change suggest that these effects, though significant, can be substantially smaller than impacts of market conditions when considering adaptation intensification. It appears as though basic underlying problems of underdeveloped markets provide the primary impetus for adaptation, even in the context of climate change.

The overall implications of our findings for policy are something of a good news/bad news story. The good news is that there seem to be some areas (such as access to weather information) that could foster adaptation across scales, particularly with respect to intensity adaptation. But the bad news is, that generalizable results, to support policy prescriptions at broad scales, are difficult to come by, particularly at the global scale. Nonetheless, there may be generalizable results that are more evident at regional or country levels. Moreover, the understanding of heterogeneity of among local determinants of adaptation is also of importance, to temper expectations of broad-based policies. Moreover, such local information can provide a wealth of information to non-profit organizations committed to support agricultural activities in these poor countries.

### Implications for further research

The results presented in this paper are largely exploratory, in that we consider a large numbers of drivers across a large numbers of scales. Complementary studies to this analysis would include many more site, country and regional comparisons. Studies at lower scales would allow for a better understanding of the specific decision making processes that may be occurring. For example, our results indicate that individual drivers of adaptation can increase either adaptive diversification or specialization, depending on local circumstances. Understanding why a given determinant can cause differing results with respect to intensification could be important to policy makers and donor agencies.

Our study was also somewhat limited in our ability to identify generalizability at various scales. Though we were able to compare global and local scales, policy-relevant information about drivers of adaptation is likely more valuable at national scales. Data sets that contained more sites within a given country could potentially yield such information.

Finally, we have investigated differences between two dimensions of adaptation (i.e. intensity and diversification). When one considers the ways in which individuals and households can adapt, there are clearly many ways that these phenomena could be conceptualized and measured. Further research into more complete ways of measuring adaptation will likely be required for better understandings.

## Supporting information

S1 Table Categories and descriptive statistics of changes in farming practices. Households responses for the questions about changes in farming practices were capture by binary indicators (e.g. response = 1 for yes, “stopped using manure/compost”). Therefore, the mean represents the proportion of the households in the sample that implemented the change.
(DOCX)

S2 Table Descriptive statistics of elements of adaptive capacity and reasons for change. Detailed descriptions for each variable are available from CCAFS Baseline Household Level Questionnaire [41].
(DOCX)

S3 Table Region, site, and crop fixed-effects estimates. (as part of regression models presented in Table 3).
(DOCX)
