# Supplementary material for: Diversification and intensification of agricultural adaptation from global to local scales
Source: PLoS One. 2018 May 4;13(5):e0196392. doi: 10.1371/journal.pone.0196392 (PMC5935394; doi:10.1371/journal.pone.0196392)
Supplement: S1 Table — Households responses for the questions about changes in farming practices were capture by binary indicators (e.g. response = 1 for yes, “stopped using manure/compost”). Therefore, the mean represents the proportion of the households in the sample that implemented the change. (DOCX) [file pone.0196392.s001.docx]

**S1 Appendix. Categories and descriptive statistics of changes in farming practices**

|  | Mean | Standard Deviation |
| --- | --- | --- |
| ***Category 1: Crop management*** | ***0.842*** | ***0.365*** |
| Introduced any new crop (over some time) | 0.350 | 0.477 |
| Are you testing any new crop (still not sure about) | 0.094 | 0.292 |
| Stopped growing a crop (totally) | 0.471 | 0.499 |
| Stopped growing a crop (in one season) | 0.229 | 0.420 |
| Introduced intercropping | 0.446 | 0.497 |
| Introduced rotations | 0.234 | 0.424 |
| Earlier planting | 0.269 | 0.443 |
| Later planting | 0.171 | 0.377 |
| Started using or using more pesticides/herbicides | 0.395 | 0.489 |
| Stared using integrated pest management | 0.043 | 0.203 |
| Started using integrated crop management | 0.035 | 0.184 |
| ***Category 2: Changing crop varieties*** | ***0.824*** | ***0.380*** |
| Introduced new variety of crops | 0.731 | 0.443 |
| Planting higher yielding variety | 0.625 | 0.484 |
| Planting better quality variety | 0.458 | 0.498 |
| Planting pre-treated/improved seed | 0.348 | 0.476 |
| Planting shorter cycle variety | 0.391 | 0.488 |
| Planting longer cycle variety | 0.158 | 0.365 |
| Planting drought tolerant variety | 0.192 | 0.394 |
| Planting flood tolerant variety | 0.063 | 0.242 |
| Planting salinity-tolerant variety | 0.019 | 0.135 |
| Planting toxicity-tolerant variety | 0.005 | 0.067 |
| Planting disease-resistant variety | 0.209 | 0.406 |
| Planting pest-resistant variety | 0.162 | 0.369 |
| Testing a new variety | 0.123 | 0.329 |
| Stopped using a variety | 0.486 | 0.500 |
| ***Category 3: Soil, water and land management*** | ***0.820*** | ***0.384*** |
| Expanded area | 0.474 | 0.499 |
| Reduced area | 0.406 | 0.491 |
| Started irrigating | 0.117 | 0.322 |
| Stopped irrigating | 0.010 | 0.098 |
| Stopped burning | 0.093 | 0.290 |
| Introduced crop cover | 0.051 | 0.220 |
| Introduced micro-catchments | 0.033 | 0.179 |
| Introduced/built ridges or bunds | 0.086 | 0.280 |
| Introduced mulching | 0.064 | 0.245 |
| Introduced terraces | 0.050 | 0.219 |
| Introduced stone lines | 0.019 | 0.138 |
| Introduced hedges | 0.044 | 0.205 |
| Introduced contour ploughing | 0.048 | 0.215 |
| Introduced improved irrigation (water efficiency) | 0.106 | 0.308 |
| Introduced improved drainage | 0.024 | 0.152 |
| Introduced tidal water control management | 0.017 | 0.128 |
| Introduced mechanized farming | 0.270 | 0.444 |
| Earlier land preparation | 0.388 | 0.487 |
| Started using or using more mineral/chemical fertilizers | 0.532 | 0.499 |
| Started using manure/compost | 0.339 | 0.474 |
| Stopped using manure/compost | 0.064 | 0.245 |

Households responses for the questions about changes in farming practices were capture by binary indicators (e.g. response =1 for yes, “stopped using manure/compost”). Therefore, the mean represents the proportion of the households in the sample that implemented the change.
