# Supplementary material for: Diversification and intensification of agricultural adaptation from global to local scales
Source: PLoS One. 2018 May 4;13(5):e0196392. doi: 10.1371/journal.pone.0196392 (PMC5935394; doi:10.1371/journal.pone.0196392)
Supplement: S2 Table — Detailed descriptions for each variable are available from CCAFS Baseline Household Level Questionnaire [41]. (DOCX) [file pone.0196392.s002.docx]

**S2 Appendix. Descriptive statistics of elements of adaptive capacity and reasons for change**

| **Variable** | **Description** | **Mean** | | **Standard Deviation** | |  |
| --- | --- | --- | --- | --- | --- | --- |
| **Access to Information & Human Capital** | | | | | |  |
| *Access to weather information* | 1 if any "Yes" to question "Did you receive any information?" | 0.742 | | 0.438 | |  |
| *Membership in farming association(s)* | 1 if "Yes" to question "Do you belong to a group(s) doing the following activities?" | 0.354 | | 0.478 | |  |
| *Education – primary* | 1 if the highest level of education attained by any household member is primary | 0.377 | | 0.485 | |  |
| *Education – secondary* | 1 if the highest level of education attained by any household member is secondary | 0.330 | | 0.470 | |  |
| *Education - post-secondary* | 1 if the highest level of education attained by any household member is post-secondary | 0.194 | | 0.396 | |  |
| **Finance** | | | | | |  |
| *Access to agricultural credit* | 1 if "Yes" to question "In the last 12 months did you get any credit for agricultural activities?" | 0.147 | | 0.354 | |  |
| *Bank account* | 1 if "Yes" to sub-question (y) of question "Which of the following items does your household own…?" | 0.312 | | 0.463 | |  |
| *Cash from the government* | 1 if "Yes" to question "Any cash income during the last 12 months?" with source from projects/government | 0.320 | | 0.467 | |  |
| *Income from non-farm employment* | 1 if "Yes" to question "Any cash income during the last 12 months?" with source from employment on someone else's farm, other paid employment or business other than farm products | 0.718 | | 0.450 | |  |
| *Income from renting out land or machinery* | 1 if "Yes" to question "Any cash income during the last 12 months?" with source from renting out machinery/land | 0.144 | | 0.351 | |  |
| **Assets** | | | | | |  |
| *Count of household assets* | Count of assets owned by a household (summation of 1s where "Yes" to question "Which of the following items does your household own…?") | 3.123 | | 2.522 | |  |
| *Livestock* | 1 if "Yes" to question "…which of the following did you produce from your own farm...?" *w.r.t.* large/small livestock | 0.864 | | 0.343 | |  |
| *Motorcycle* | 1 if "Yes" to question "Which of the following items does your household own…?") *w.r.t.* motorcycle | 0.147 | | 0.354 | |  |
| *Car or truck* | 1 if "Yes" to question "Which of the following items does your household own…?") *w.r.t.* car or truck | 0.032 | | 0.175 | |  |
| *Boat* | 1 if "Yes" to question "Which of the following items does your household own…?") *w.r.t.* boat | 0.008 | | 0.091 | |  |
| **Farm & Household Characteristics** | | | | | |  |
| *Running water* | 1 if "Yes" to question "Which of the following structures/utilities does your household have?" *w.r.t.* running/tap water | 0.156 | | 0.362 | |  |
| *Storage facility for crops* | 1 if "Yes" to question "Which of the following structures/utilities does your household have?" *w.r.t.* improved storage facility for crops | 0.213 | | 0.410 | |  |
| *Planted trees* | 1 if household has planted at least one tree on his farm | 0.378 | | 0.485 | |  |
| *Household size* | Number of people living in a household | 7.143 | | 5.340 | |  |
| *Household is female-headed* | 1 if the gender of household head is female | 0.100 | | 0.300 | |  |
| S2 Appendix (cont’d) | | | | | |  |
| **Farming & Crisis Experience** | | | | | |  |
| *Farming experience is at least 10 years* | 1 if "Yes" to question "Have you or your family been farming or keeping animals or fish in this locality for 10 years or more?" | | 0.927 | | 0.260 | |
| *Experienced climate crisis in the last 5 years* | 1 if "Yes" to question "Have you faced a climate related crisis in the last 5 years?" | | 0.730 | | 0.444 | |
| **Stated Reasons For Changes** | | | | | | |
| *Market conditions* | 1 if any crop is recorded for question "Why you have made these changes and to which crops?" *w.r.t.* Markets | | 0.713 | | 0.452 | |
| *Climate variability* | 1 if any crop is recorded for question "Why you have made these changes and to which crops?" *w.r.t.* Climate | | 0.482 | | 0.500 | |
| *Pests and disease* | 1 if any crop is recorded for question "Why you have made these changes and to which crops?" *w.r.t.* Pests & diseases | | 0.323 | | 0.468 | |
| *Government/NGO intervention* | 1 if any crop is recorded for question "Why you have made these changes and to which crops?" *w.r.t.* Projects etc. | | 0.108 | | 0.310 | |
| *Labor availability* | 1 if any crop is recorded for question "Why you have made these changes and to which crops?" *w.r.t.* Labour | | 0.404 | | 0.491 | |
| *Land productivity* | 1 if any crop is recorded for question "Why you have made these changes and to which crops?" *w.r.t.* Land | | 0.507 | | 0.500 | |

Detailed descriptions for each variable are available from CCAFS Baseline Household Level Questionnaire [38].

|  |  |
| --- | --- |
