# Supplementary material for: Diversification and intensification of agricultural adaptation from global to local scales
Source: PLoS One. 2018 May 4;13(5):e0196392. doi: 10.1371/journal.pone.0196392 (PMC5935394; doi:10.1371/journal.pone.0196392)
Supplement: S3 Table — (as part of regression models presented in Table 3). (DOCX) [file pone.0196392.s003.docx]

**S3 Appendix. Region, Site, and Crop Fixed-Effects Estimates**

(as part of regression models presented in Table 3)

|  | Adaptation  Intensification  Model | Adaptation  Diversification  Model |
| --- | --- | --- |
| **REGIONAL FIXED EFFECTS (Base case = Central America)** | | |
| region==East Africa | -3.609*** | -0.284 |
|  | (0.853) | (0.194) |
| region==West Africa | 2.680*** | -0.453*** |
|  | (0.640) | (0.164) |
| region==South Asia | -5.673*** | -0.370* |
|  | (0.689) | (0.206) |
| **SITE FIXED EFFECTS (Base case = MZ02, Mozambique)** | | |
| siteid==BA01 | 3.768*** | -0.407* |
|  | (0.967) | (0.208) |
| siteid==BA02 | 2.754*** | -0.148 |
|  | (0.730) | (0.191) |
| siteid==BA03 | 2.450** | 0.089 |
|  | (1.030) | (0.219) |
| siteid==BA04 | 3.429*** | -0.290 |
|  | (0.844) | (0.219) |
| siteid==BA05 | 3.725*** | -0.182 |
|  | (0.872) | (0.220) |
| siteid==BA06 | 2.033*** | -0.409* |
|  | (0.763) | (0.212) |
| siteid==BA07 | 3.809*** | -0.407* |
|  | (0.767) | (0.214) |
| siteid==BF01 | -0.441 | 1.174*** |
|  | (0.772) | (0.199) |
| siteid==CR04 | -1.296* | -0.180 |
|  | (0.773) | (0.159) |
| siteid==ET01 | 0.676 | 0.370** |
|  | (0.992) | (0.187) |
| siteid==GH01 | -2.502*** | -0.084 |
|  | (0.430) | (0.147) |
| siteid==IN08 | 1.145** | 0.252 |
|  | (0.470) | (0.183) |
| siteid==IN09 | 2.063*** | 0.402** |
|  | (0.454) | (0.162) |
| siteid==IN10 | 2.015*** | 0.213 |
|  | (0.450) | (0.167) |
| siteid==IN11 | 3.631*** | 0.271* |
|  | (0.473) | (0.158) |
| siteid==IN12 | 5.154*** | -0.232 |
|  | (0.508) | (0.177) |
| siteid==IN13 | 3.889*** | 0.394** |
|  | (0.426) | (0.160) |
| siteid==IN14 | 4.094*** | 0.018 |
|  | (0.359) | (0.168) |
| siteid==IN16 | 5.517*** | -0.034 |
|  | (0.342) | (0.163) |
| siteid==IN17 | 5.595*** | -0.308* |
|  | (0.467) | (0.159) |
| siteid==KE01 | 4.715*** | 0.265 |
|  | (0.823) | (0.180) |
| siteid==KE02 | 6.451*** | 0.076 |
|  | (0.704) | (0.178) |
| siteid==MA01 | -5.059*** | 0.278** |
|  | (0.552) | (0.141) |
| siteid==MZ01 | 5.945*** | 0.248 |
|  | (0.825) | (0.171) |
| siteid==NC01 | 2.836*** | 0.099 |
|  | (0.536) | (0.147) |
| siteid==NC02 | -2.816*** | 0.079 |
|  | (0.613) | (0.123) |
| siteid==NE01 | 2.082*** | 0.160 |
|  | (0.349) | (0.114) |
| siteid==NE02 | 1.527*** | 0.411* |
|  | (0.338) | (0.220) |
| siteid==NE03 | 2.429*** | 0.407*** |
|  | (0.284) | (0.136) |
| siteid==NE04 | 2.135*** | 0.522*** |
|  | (0.514) | (0.098) |
| siteid==NI01 | -1.336*** | 0.328** |
|  | (0.432) | (0.138) |
| siteid==TZ01 | 5.944*** | 0.468** |
|  | (0.790) | (0.203) |
| siteid==UG01 | 4.278*** | 0.624*** |
|  | (0.750) | (0.173) |
| siteid==UG02 | 4.093*** | 0.699*** |
|  | (0.791) | (0.208) |
|  |  |  |
|  |  |  |
| **CROP FIXED EFFECTS (Base case = all other non-top 15 crops)** | | |
| Maize | 1.489*** | -0.187*** |
|  | (0.164) | (0.061) |
| Rice | 2.150*** | 0.039 |
|  | (0.202) | (0.053) |
| Wheat | 3.063*** | -0.098 |
|  | (0.448) | (0.087) |
| Beans | 1.152*** | -0.179*** |
|  | (0.145) | (0.059) |
| Mustard | 0.898*** | -0.107* |
|  | (0.151) | (0.055) |
| Banana | 0.762*** | -0.128** |
|  | (0.154) | (0.052) |
| Sorghum | 1.018*** | -0.067 |
|  | (0.222) | (0.065) |
| Millet | 0.099 | -0.091 |
|  | (0.284) | (0.080) |
| Cowpeas | 0.922*** | -0.085 |
|  | (0.266) | (0.064) |
| Potatoes | 0.793*** | -0.071* |
|  | (0.156) | (0.040) |
| Cassava | 0.723*** | -0.083 |
|  | (0.173) | (0.053) |
| Peanuts | 0.776*** | -0.074 |
|  | (0.197) | (0.059) |
| Coffee | 0.683*** | -0.114** |
|  | (0.154) | (0.056) |
| Rice - Aman | 3.689*** | 0.030 |
|  | (0.679) | (0.089) |
| Lentils | 1.060*** | -0.090 |
|  | (0.188) | (0.079) |
| The table reports the effects of regions, sites, and crops obtained from the estimation of the global models (effects of elements of adaptive capacity and reasons for changes are reported in Table 3). Like in Table 3, for the adaptation intensification count model, estimates represent marginal effects. Estimates of the adaptation diversification model represent percentage changes in the HHI index. Central America and MZ02, Mozambique respectively are the baseline region and site. They are selected due to the lowest average count of activities among households. Robust standard errors clustered at the village level are in parentheses. Significance levels are: * p<0.1; ** p<0.05; *** p<0.01 | | |
